# Hospital-Based Prevalence, Electroencephalogram (EEG), and Neuroimaging Correlation in Seizures Among Children in Odisha, India

**DOI:** 10.7759/cureus.21103

**Published:** 2022-01-11

**Authors:** Swarnalata Das, Pragyan Paramita, Natabar Swain, Riya Roy, Santwana Padhi, Soumini Rath, Sanjukta Mishra, Nirmal K Mohakud

**Affiliations:** 1 Pediatrics, Kalinga Institute of Medical Sciences, Bhubaneshwar, IND; 2 Pediatrics, Kalinga Institute of Medical Sciences, Bhubaneswar, IND; 3 Bioinnovation, Technology Business Incubator, Kalinga Institute of Industrial Technology, Deemed to be University, Bhubaneswar, IND; 4 Technology Business Incubator, Kalinga Institute of Industrial Technology, Deemed to be University, Bhubaneswar, IND; 5 Neonatology, Kalinga Institute of Medical Sciences, Bhubaneswar, IND; 6 Biochemistry, Kalinga Institute of Medical Sciences, Bhubaneswar, IND; 7 Pediatric Medicine, Kalinga Institute of Medical Sciences, Bhubaneswar, IND

**Keywords:** neuroimaging, generalized seizure, children, mri, eeg, febrile seizures

## Abstract

Background: Febrile seizures are very common in pediatric practice. We need to differentiate between febrile seizures and other seizures due to central nervous system (CNS) infection by various means of investigation. Though approximately 30% of patients with febrile seizure have later epilepsy and the risk is around 20% even if electroencephalogram (EEG), and neuro-imagings are normal. But data regarding this is laking in developing countries like India.

Aim: The primary objective of this study is to determine the hospital-based prevalence among various types and etiologies of seizures in children admitted to the pediatric department in a teaching hospital of a developing country, India. Besides, the different types of seizures were correlated with the EEG and neuroimaging findings along with the acute onset of seizures among children.

Methods: In this prospective observational study, children from two months to 15 years of age admitted to the Pediatrics Department, KIMS, Bhubaneswar in India between September 2017 and September 2019 were taken. The patients having seizures were included in the study based on the inclusion criteria. Neurological and systemic examinations of the children were recorded and the neuroimaging reports were analyzed.

Results: A total of 19,553 patients aged two months to 15 years were admitted during the study period. Of that, 1,192 cases were diagnosed with febrile and unprovoked seizures. It was observed that the hospital-based prevalence of seizures among children in Odisha was 6%. Besides, it is found that generalized seizure disorder was the most common among the children. It was found that abnormal EEG, magnetic resonance imaging (MRI), and computed tomography (CT) brain in 60% (202/340), 49% (113/230), and 47% (136/288) of cases, respectively. MRI is a better modality of investigation in partial seizure cases 22 (64%) to detect CNS abnormality. Also, MRI of the brain is better in evaluating CNS abnormality in complex febrile cases 4 (31%) than CT brain (0%).

Conclusion: The study concluded that EEG must be the standard modality of test for patients' diagnosis of seizure in children with seizures. CT/MRI scan can give a better supplement to the results but MRI findings are more accurate in cases of complex febrile seizures.

## Introduction

Abnormal excessive/synchronous neuronal activity in the brain causes the transient occurrence of involuntary contraction of muscles called seizures, which is an important cause for hospital admissions of children. Approximately 30% of patients with febrile seizures have later epilepsy, and the risk is around 20% even if electroencephalogram (EEG), and neuroimaging are normal [1].

These seizures signify structural, inflammatory, or metabolic disorders of the brain like meningitis, encephalitis, acute stroke, or brain tumor. The prognosis depends on the underlying cause, its reversibility or treatability, and the likelihood of developing epilepsy. Some of the common types of acute seizures in children are neonatal seizures caused due to infections, metabolic disorders, febrile seizures, meningitis, viral encephalitis, cerebral malaria, and epilepsy [2]. Central nervous system (CNS) infections are the main cause of seizures and acquired epilepsy in the developing world [2]. The incidence is highest in children less than three years of age and one-fifth of total children with unprovoked seizures may develop epilepsy [3,4]. Febrile seizures affect 3% of all children below six years of age. About 4%-10% of children have an experience of at least one episode of seizure in the first 16 years of life [3].

There is a need to differentiate between febrile seizures and other seizures. Many investigations are there to diagnose the etiology of the first episode of seizure and a combination of investigations is used to reach the diagnosis. We try to find out the single most investigation needed to yield the highest correlation with seizure, as the data regarding this is lacking in developing countries like India. Children with seizures must get a proper diagnosis at the earliest for better management and a good prognosis [5]. This prospective observational study was conducted to investigate the socio-demographic profile, clinical characteristics, hospital-based prevalence, and correlation of EEG and neuroimaging in children under 15 years with seizures in Odisha state.

## Materials and methods

This prospective observational study was undertaken among children aged two months to 15 years with seizures, admitted to the Department of Pediatrics, KIMS from September 2017 to September 2019. All types of seizures (generalized convulsion with/without neurologic deficit, Focal convulsion with/without neurologic deficit, seizure due to CNS infections, simple and complex febrile seizures, status epilepticus, epileptic seizure, post-hypoxic-ischemic encephalopathy [HIE] sequelae, inborn errors of metabolism, intracranial space-occupying lesions, congenital malformation, demyelination, and neurodegenerative disorders) except neonatal seizures were included in the study. Patients with primary complaints of seizure were evaluated by understanding the detailed history, and conducting a thorough clinical examination and investigations as per the case record proforma. Ethical clearance from IEC KIMS (KIMS/KIIT/IEC/81/2017/15.9.2017) and consent from the parents was obtained prior to enrolment of the cases.

Case record proforma

The case records proforma required basic personal information like name, sex, age, address, contact details, date of birth, history of any prior/present illness. Type, duration, frequency, and aura of the seizure were recorded along with any associated symptoms. The developmental history, family history, and socioeconomic status was recorded according to the Modified Kuppuswamy Scale of nutritional history. The general physical examination and a detailed neurological and systemic examination of the patients were conducted. 

Prior to initiation of therapy, blood was collected for hematological and biochemical examination. To estimate the blood glucose from the finger-prick sample, glucometer and dextrostix strip were used. The lumbar puncture was done in selected cases as per the clinical indication and prior to initiation of antibiotic therapy. Cerebrospinal fluid (CSF) pressure, color was noted and a sample was sent for cytological, biochemical, and bacteriological examination. Fundoscopy was done in all the cases.

The cut-off values of biochemical abnormalities were ( hypoglycemia if blood glucose levels were less than 50 mg/dl, hypocalcemia if the serum calcium level was less than 8 mg/dL, and hyponatremic seizure, if the serum sodium value was less than 130 meq/L) taken as defined [6]. Pyogenic meningitis was diagnosed if there is a decreased CSF glucose level (<40 mg/dL), raised CSF protein (>40 mg/dL), and predominance of polymorphonuclear cells (>5 leukocytes) [7]. Convulsion due to encephalitis was considered in a CSF report of variable protein levels with a minimal cellular response and normal glucose level. In most of the cases, EEG and computed tomography/magnetic resonance imaging (CT/MRI) of the brain were done.

EEG recordings

Pre-requisites

Some patients were advised not to sleep overnight. Sleep was induced with syrup trichlorophus (0.5 mL/kg body weight) orally or IV lorazepam (0.1 mg/kg body weight) injection as a starter dose.

Methods of Tracing

EEG was performed on all the subjects, by using a 16-channel EEG machine. A total of 21 electrodes were placed using bentonite electrode paste. Before applying the electrodes, the contact surface of the scalp was thoroughly washed with spirit to make perfect contact. It was adjusted so as to generate an input signal of 50 microvolts resulting in a deflection of 7 millimeters.

The standard paper speed of recording was 30 mm/sec. EEG was performed within 72 hours of unprovoked and provoked seizures. Each recording was obtained with a minimum duration of 20-30 minutes and the electrode was positioned over the scalp according to the international 10-20 system. The recording was done in both awake and sleep states. Provocative stimuli like hyperventilation, photic stimulation were given for 3 minutes each when required. EEG was analyzed by a clinical neurologist from the department of Neurology, KIMS, Bhubaneswar.

Study of EEG Tracing

The standard recording speed is 30 mm/sec, calibration 50 microvolt (deflection of 7mm). The first eight channels show changes in the right hemisphere and the last 8 channels show changes in the left hemisphere.

Frequency of Rhythm

EEG frequencies are classified into the following four ranges as Beta Rhythm: 13 Hertz/second (Hz/sec), Alpha Rhythm: 8-13 Hz/sec, Theta Rhythm: 4-8 Hz/sec, Delta Rhythm:<4 Hz/sec Epileptiform activity are asymmetry with a sharp wave, the asymmetry with a sharp wave and spike, abnormal background with the sharp wave, abnormal background with the sharp wave and spike, sharp wave alone, spike alone, sharp wave and spike, and hypsarrhythmia. EEG was called abnormal when there is an intermittent or persistent focal slowing seen consistently over one head region, or persistent, unvarying, and unreactive focal or generalized slow-wave activity [8].

CT of the brain was performed with help of a third-generation CT scan machine (multi-detector CT). Cranial MRI was performed on patients with an abnormal EEG, and all undiagnosed seizures. MRI scan was carried out in a 1.5 Tesla magnet. Abnormal neuroimaging was categorized into cerebral atrophy, dilated ventricle, calcification, cerebral edema, periventricular leukomalacia, encephalomalacia, cystic lesion, vascular malformation, and stroke. Though all patients of this study group were advised for neuro-imaging, few had their reservations for the investigation.

Statistical analysis

The proportional differences of recorded data were measured by Chi-square analysis. All inferences were drawn using standard statistical software(Strata 13.1, StataCorp, College Station, TX, USA). P-value of <0.05 was considered to be statistically significant.

## Results

**Figure 1:**
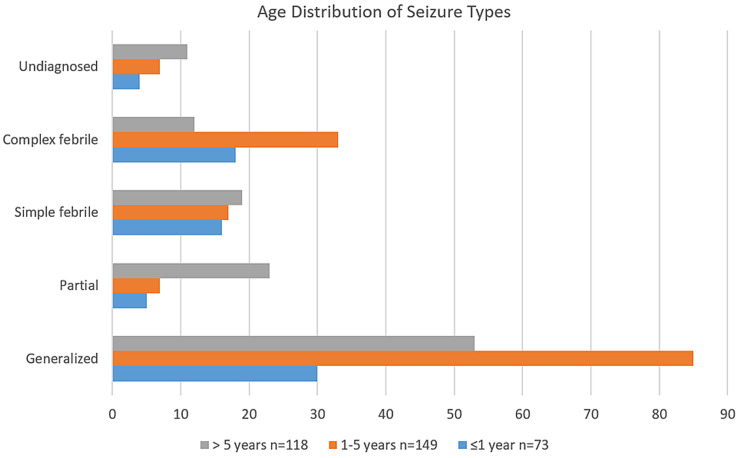
Distribution of seizure among children two months to 15 years

The total number of cases admitted in the pediatric ward during the study period was 19,553 and 1,192 cases presented with both febrile, unprovoked seizures, and neonatal seizures. The number of cases of seizure included in this study was 340. There was a male 235 (69%) predominance (M:F; 2.2:1). The hospital-based prevalence of seizures in children was 6% (1,192/19,553). The majority of children with seizures are in age group1-5 years 149 (43.8%) (Figure 1).

**Figure 2:**
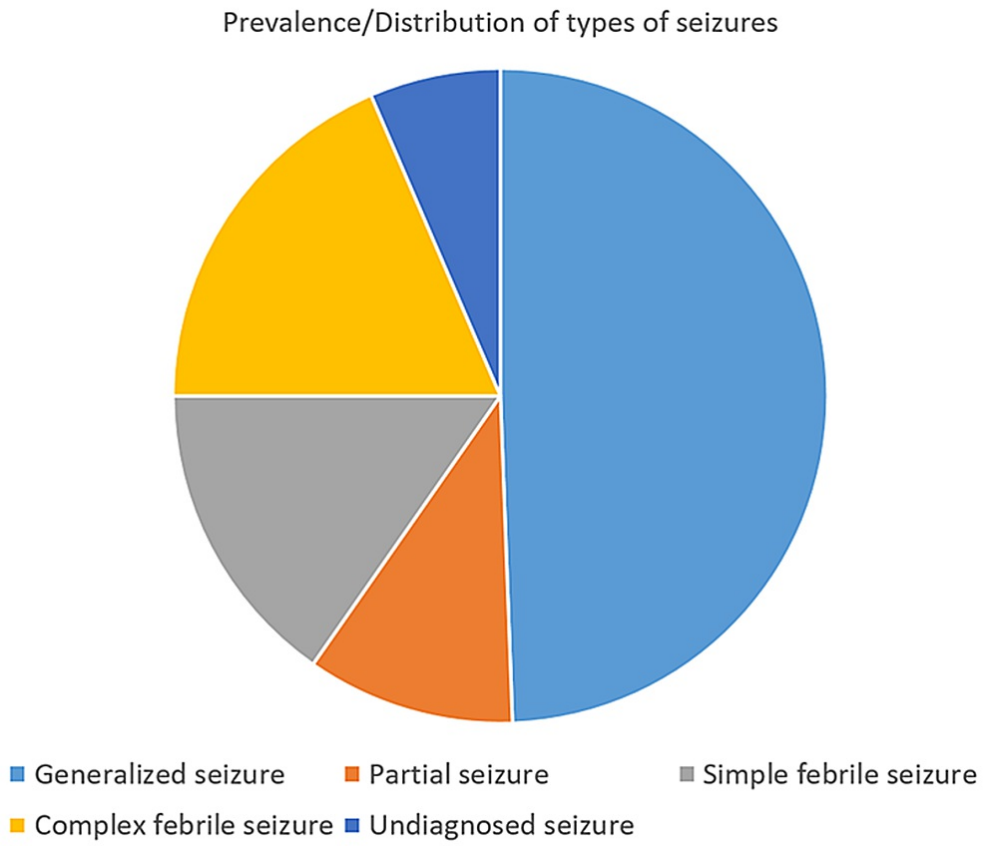
Distribution of seizure types among the children with convulsion (two months to 15 years)

Similarly, generalized seizure disorder 165 (48.5%) was the most common in children (Figure 2).

The prevalence of different etiologies of seizures in children were shown in Table 1. 

**Table 1:** The prevalence of different etiologies of seizures in children two months to 15 years of age, except simple febrile seizure cases in KIMS, Odisha

Diagnosis	Generalized seizure	Partial seizure	Complex febrile seizure	Undiagnosed seizure	Total n = 288
Infective Group	20	7	0	0	27 (9%)
Epilepsy Group	94	16	0	0	110 (38%)
Cerebral palsy	47	8	0	0	55 (19%)
Cerebrovascular accident	2	0	0	0	2 (1%)
Intracranial space-occupying lesion	2	3	0	0	5 (2.3%)
Congenital malformation	3	1	0	0	4 (2%)
Complex febrile seizure	0	0	63	0	63 (22%)
Undiagnosed seizure	0	0	0	22	22 (7%)
Total	168 (59%)	35 (12%)	63 (22%)	22 (7%)	288 (100%)

Non-infectious is the predominant etiology of seizure accounting for 291 (84%) of cases. Among the non-infectious cases, the idiopathic epilepsy group constitutes the highest number 110 (38%). It was found that 52 (15.3%) had simple febrile seizures. It was seen that 60% of the total cases in our study had an abnormal EEG recording. Abnormal EEG recordings are very characteristic in generalized seizures 195 (95%). However, no cases of abnormal EEG were found among children with simple febrile seizures (Table 2).

**Table 2:** EEG findings in different seizure types in children two months to 15 years of age, KIMS, Odisha

Seizure	Normal EEG, n=138	Abnormal EEG, n=202	Total (%), n=340
Generalised	9 (5%)	159 (95%)	168 (50%)
Partial	7 (20%)	28 (80%)	35 (10%)
Simple febrile	52 (100%)	0	52 (15%)
Complex febrile	49 (78%)	14 (22%)	63 (18%)
Undiagnosed	21 (95%)	1 (5%)	22 (7%)
Total (%)	138 (40%)	202 (60%)	340 (100%)

When we had observed the CT scan of the brain in seizure patients, it was found to have no significant difference among various seizure groups and normal CT brain in 152 (53%) compared to abnormal in 136 (47%) children (Table 3).

**Table 3:** CT scan abnormality in different seizure types in children two months to 15 years of age, KIMS, Odisha

Seizure	Normal CT, n=152	Abnormal CT, n=136	Total (%), n=288
Generalized	61 (36%)	107 (64%)	168 (58%)
Partial	8 (23%)	27 (77%)	35 (12%)
Complex febrile	63 (100%)	0	63 (22%)
Undiagnosed	20 (91%)	2 (9%)	22 (8%)
Total (%)	152 (53%)	136 (47%)	288 (100%)

On the evaluation of MRI in these seizure children, it was found that MRI is a better diagnostic tool in partial seizure cases 22 (64%). Also, MRI of the brain is better in evaluating CNS abnormality in complex febrile cases 4 (31%) than CT brain having none. Further, among the children with undiagnosed seizures had normal MRI findings in all cases 22 (100%). About 51% of them had normal and 49% had abnormal MRI findings (Table 4).

**Table 4:** MRI findings in different types of seizures in children two months to 15 years of age, KIMS, Odisha

Seizure	Normal MRI, n=117	Abnormal MRI, n=113	Total, n=230
Generalized	72 (45%)	87 (55%)	159 (69%)
Partial	13 (36%)	22 (64%)	35 (15%)
Complex febrile	10 (69%)	4 (31%)	14 (6%)
Undiagnosed	22 (100%)	0	22 (10%)
Total	117 (51%)	113 (49%)	230 (100%)

It was found that MRI of the brain is better correlated with abnormal EEG in seizure children. Almost 60% (137) of children having seizures had abnormal MRI compared to 88% (202) children with abnormal EEG (Table 5).

**Table 5:** Association of MRI and EEG findings among children presenting with seizures

MRI	Normal EEG, n=28	Abnormal EEG, n=202	Total, n=230
Normal	13 (46%)	80 (40%)	93 (40%)
Abnormal	15 (54%)	122 (60%)	137 (60%)
Total	28 (12%)	202 (88%)	230 (100%)

## Discussion

It is essential to gather more and more data on seizure disorders in children so that the timely and appropriate treatment of seizures will yield a better prognosis. In this study, we found the hospital-based prevalence of seizures in children was 6% and the majority are in the age group 1-5 years. Besides, it is found that generalized seizure disorder was the most common among children. MRI should be the investigation modality of choice to find out CNS abnormalities in seizure disorders in children. Many studies have been conducted to determine the etiologies of convulsions in childhood [9,10]. This particular study was to correlate the clinico-demographic pattern of seizures, etiology, neuroimaging, and EEG abnormality, and the prevalence was found to be 6% in a tertiary care hospital in Odisha. However, a crude prevalence of 5.35/1,000 children is there [11]. In the present study, there was male preponderance also reported in various other studies [10,12]. Male predominance was attributed to their greater liabilities to congenital cerebral defects and birth injuries, which could have lowered the convulsive threshold of the brain [13].

The maximum number of cases 149 (43.8%) belonged to 1-5 years age group, 21% were below one year of age group and 35% of cases were >5 years of age. Similar observation reported that seizures began before age of one year in 32% of cases [13,14].

A maximum number of cases in our study, 159 (47%) belonged to middle-class status and 43% belonged to the lower class. Generalized tonic-clonic seizures were the most commonly seen in 154 (46%) cases followed by generalized absence seizures seen in eight (2%) cases. This observation was similar to previously conducted studies, that generalized tonic-clonic seizure was the most common (58.3%) seizure type in all age groups and in both sexes [9,10]. Out of 149 cases in the 1-5-year age group, 57% had a generalized seizure, 22% had a complex febrile seizure and 11% had a simple febrile seizure. In 73 cases <1 year age group, 41% had a generalized seizure, 25% had a complex febrile seizure. Among 118 (35%) cases in the >5-year age group, 45% had generalized and 19% had a partial seizure (Figure 2). There was a similar observation in a study revealing generalized tonic seizures were 54.5%, 62.8%, and 53.3% in the age group of <1 year, 1-5 years, and > 5 years, respectively [15].

Of the children having a generalized seizure, 95 (62%) presented with fever, 30% had vomiting, 28% had altered sensorium, 18% had meningeal signs and 11% had signs of raised intracranial tension. The most common clinical features were fever, altered sensorium, and vomiting which was consistent with the finding in western Nepal and east and south India [16-19]. About 95% of cases presented generalized seizure with an abnormal EEG, 80% of the partial seizure cases had abnormal EEG. In the cases with simple febrile seizures, 95% of undiagnosed seizures and 78% of complex febrile seizures had a normal EEG. In various studies, abnormal EEG was reported in 81% of partial seizure cases and 78% of generalized seizures [20,21]. It was seen that 73% of patients with partial seizures and 76.9% with generalized seizures had an abnormal EEG [22]. These observations were coherent with research was done using EEG findings of children with seizures and epilepsy [23,24].

Our study reflected abnormal EEG in the complex febrile seizures in 2% cases, similar to another study on febrile seizures in children [25-27]. Another study reported that 20% of undetermined seizure cases had an abnormal EEG [28]. In our study, 5% of undiagnosed cases and 159 cases of generalized seizure had an abnormal EEG. It is important to note that 100% of generalized absence seizure and infantile spasm cases had an abnormal EEG. 66.7% generalized tonic-clonic, 62.5% partial seizure, 100% generalized absence, and 100% infantile spasm cases were also reported previously [15].

Out of 288 cases in this study, 47% had an abnormal CT and 53% had a normal CT (Table 2). About 77% of partial seizure cases and 64% of generalized seizures had an abnormal CT. Of all the cases with complex febrile seizures, 91% of undiagnosed seizures and 36% of generalized seizure cases had normal CT scans of the brain. A similar observation was seen that abnormal CT scans in 73% of patients suffered from partial seizure [10,29,30].

On studying the association of CT and EEG findings, it is found that 202 cases with abnormal EEG recording had the chance of finding an abnormal CT was in 111 (55%) cases. Also, among 86 normal EEG cases, the chance of finding an abnormal CT was in 29% of the patients. This result was similar to a study in western Rajasthan where 57.8% and 28.5% had a chance of finding abnormal CT scans within abnormal and normal EEG, respectively [27].

The current study showed 49% had an abnormal MRI scan. Abnormal MRI was found in 55% of generalized seizures, 64% of partial seizure cases, and 31% of complex febrile seizure cases. A study reported 32.5% MRI abnormality in children with newly diagnosed seizures [31]. Out of 87 patients with generalized seizure who had an abnormal MRI, 42 had periventricular leukomalacia, 22 had cerebral atrophy, eight had increased signal intensity. Out of 22 patients with partial seizures, five cases had cerebral atrophy, four each had dilated ventricle with basal exudate, periventricular leukomalacia, and dilated ventricle with aqueductal stenosis. On studying the association MRI with EEG findings, out of 202 cases of abnormal EEG, 60% had abnormal MRI. And out of 28 normal EEGs, 54% had abnormal MRIs and 46% had a normal MRI. This could be due to the higher diagnostic sensitivity of MRI machines.

Our observation showed that out of 136 abnormal CTs, the chance of finding an MRI abnormality was in 70% of cases. Among 94 cases with normal CT, the chance of having a normal MRI was in 77 cases.

Limitations

The present study has its share of limitations as we have not taken outpatient cases. Also, this is a single-centered study in a private teaching hospital that may not cater to patients from a lower segment of society. Also, we are not able to do CT/MRI/ EEG in all included patients due to various reasons.

## Conclusions

The results in our study proposed EEG to be the basic test for every newly diagnosed seizure patient and further neuroimaging is to be considered in children having abnormal EEG. Abnormal EEG in partial seizures increases the probability of having abnormal neuroimaging. MRI has an advantage over CT scans in the diagnosis of complex febrile seizures. Etiology can be suspected from the EEG abnormality and should be confirmed with neuroimaging in most cases of newly diagnosed seizures.
